# Cycling Interventions Enhance Executive Function in Children with Autism via Heart Rate Variability Mediation: A Randomized Controlled Trial

**DOI:** 10.1186/s40798-026-01019-8

**Published:** 2026-04-10

**Authors:** Andy C. Y. Tse, Paul H. Lee, Eric T. C. Poon, Amy W. Y. Ho, F. H. Sun, Kean Poon, Patrick P. K. Ip, David I. Anderson

**Affiliations:** 1 Department of Sports Science and Physical Education, The Chinese University of Hong Kong, Hong Kong, China; 2 Southampton Clinical Trial Units, University of Southampton, Southampton, UK; 3 Department of Chemical Pathology, The Chinese University of Hong Kong, Hong Kong, China; 4 Department of Health and Physical Education, The Education University of Hong Kong, Hong Kong, China; 5 School of Education, University of New South Wales, Kensington, Australia; 6 Department of Paediatrics and Adolescent Medicine, The University of Hong Kong, Hong Kong, China; 7 Department of Kinesiology, San Francisco State University, San Francisco, USA

**Keywords:** Autism spectrum disorder, Executive function, Cycling, Heart rate variability, Randomized controlled trial, Children

## Abstract

**Background:**

Children with autism spectrum disorder (ASD) exhibit deficits in executive function (EF). Cycling exercise has previously shown to be effective in improving EF. However, the role of different components (e.g., spatial updating and dynamic balance) driving these positive effects remains unclear. This study examined the cognitive effect of cycling interventions with varying navigation and balance demands through a heart rate variability (HRV) mediating model.

**Methods:**

Fifty-one children with ASD (M_age_ = 8.78 years; SD = .54) were randomized into four groups: Learning to Bicycle (LTB, n = 13), Bicycle Treadmill (BT, n = 12), Cycling with Training Wheels (TW, n = 14), and Stationary Cycling (SC, n = 12). The interventions lasted for 8 sessions over 2 weeks. EF (Tower of London, TOL; Corsi Block Tapping Task, CBTT; Go/No-go, GNG; Children’s Color Trails Test, CCTT) and HRV (RMSSD) were assessed at baseline (T1), mid-intervention (T2) and post-intervention (T3).

**Results:**

All EF domains improved significantly in LTB and TW (*ps* < .001, *d* = .56–2.15) and so did HRV in LTB (*ps* < .001, *d* = 1.24). HRV partially mediated EF improvements in LTB (*β* = .35–1.62) and TW (*β* = .31–1.38), but not in BT and SC. BT showed modest to moderate EF gains (*d* = .07–.97), while SC showed minimal effects (*d* = .00–.59).

**Conclusions:**

Cycling interventions with spatial updating and dynamic balance components benefited EF the most in children with ASD, with HRV as a partial mediator. These findings provided insight into optimizing exercise interventions to address EF deficits in children with ASD.

*Trial registration* NCT07295912 (registered retrospectively on 19th Dec 2025 at https://clinicaltrials.gov/ct2/show/NCT07295912. We acknowledge that best practice is prospective registration per ICMJE guidelines).

## Key Points

1. Cycling interventions that incorporated spatial updating and dynamic balance components led to significant improvements in all executive function (EF) domains in children with autism spectrum disorder (ASD).

2. Spatial updating is the key component of exercise for inducing cognitive benefits.

3. Heart Rate Variability (HRV) is an important physiological mechanism linking cycling exercise to cognitive enhancements

## Introduction

Executive functions (EFs) encompass a set of higher-order cognitive abilities critical for goal-directed behavior, including cognitive planning, working memory, inhibition and cognitive flexibility [1]. These processes, primarily mediated by the prefrontal cortex [2], are essential for problem-solving, planning, and managing social interactions [1, 3]. However, children with Autism Spectrum Disorder (ASD) who are characterized by deficits in social communication and restricted, repetitive behaviors [4], often exhibit significant EF impairments [5]. Demetriou and colleagues showed that moderate deficits are evident across all EF domains in individuals with ASD and negatively impact their concept formation, mental flexibility, fluency, planning, response inhibition and working memory [6]. These deficits are closely associated with core symptoms in children with ASD, including social impairment, repetitive behaviors, and communication difficulties [7]. Emerging insights suggest that deficits in EF represent a promising endophenotype in ASD [6, 8].

Given the importance of EF in cognitive functions, it is necessary to search for an effective intervention to ameliorate the EF deficits in children with autism. Physical exercise has emerged as a promising, cost-effective alternative to improve EF in children with ASD [9, 10]. Recently, Tse and colleagues conducted a RCT study investigating the impact of physical exercise interventions on different EF components (i.e., planning, working memory, inhibition control and cognitive flexibility) [11]. In the study, sixty-two children with ASD were randomly assigned into three groups: learning to ride a bicycle group (n = 22), stationary cycling group (n = 20) and a control group (n = 20). Bike riding exercise was chosen because it is a popular, age-appropriate and functional activity [11]. The EF of the participants was assessed before and after the 2-week interventions. Results revealed that the bicycle learning group outperformed the stationary cycling group and control group significantly in all EF assessments after the intervention [11]. Interestingly, no significant differences were observed between the stationary cycling group and the control group in any EF measurements after the intervention period. These findings suggested that physical exertion alone did not have any effect on EF.

However, it is still unclear which component(s) of the cycling intervention plausibly drove the positive effect on EF. It is important to identify such component(s) to optimize the effects of the exercise interventions on cognitive functions for children with ASD. For example, if a particular component of the cycling intervention was shown to enhance EF in children with ASD, practitioners should incorporate such a component when designing interventions for cognitive enhancements in children with ASD. Furthermore, new interventions could also be designed to recruit that particular component more effectively. The generalizability of the findings could also be extended to children with other neurodevelopmental disorders, including attention-deficit/hyperactivity disorder (ADHD), learning disabilities, intellectual disability and cerebral palsy.

In the present study, we hypothesize that two mechanisms: (A) spatial updating (navigating the body through space) and (B) dynamic balancing (maintaining stability on a narrow base) may facilitate the positive effects of the cycling interventions on EF. These were selected based on prior evidence in healthy and older adults suggesting that spatial updating enhances memory and spatial cognition via hippocampal-prefrontal engagement [12, 13], while dynamic balance improves attention and inhibition through cerebellar-frontal circuits [14]. Other components like coordination and endurance were not prioritized as they are common to all groups (including SC) and did not differentiate effects in prior studies [11]. Studies in healthy and older adults suggested that spatial updating and balance training could enhance cognitive functions such as memory and spatial cognition [12–14] but their impact on EF in children with ASD remains unexplored. The primary objective of the present study was to determine which component(s) (i.e., spatial updating, dynamic balance or both) drive EF improvement in the cycling interventions. The neuropsychological performance of four groups: (1) learning to bicycle (LTB, recruiting A and B), (2) bicycle treadmill (BT, recruiting B), (3) cycling with training wheels (TW, recruiting A), and (4) stationary cycling (SC, recruiting neither) was compared in the present study.

Moreover, a particular interest of the present study is the underlying mechanism by which physical exercise induces changes in EF in children with ASD. It is important to understand the underlying mechanism in order to optimize the impacts of physical exercise interventions on EF in children with ASD. In children with typical development, researchers have proposed a physiological mechanism involving heart rate variability. Heart rate variability (HRV) is defined as variation in the time interval between heartbeats [15]. It is determined by interactions between the sympathetic and parasympathetic indicators of autonomic nervous system functioning [16–18]. Studies have shown that HRV, expressed as the root mean square of successive differences (RMSSD), is a reliable indicator of parasympathetic activity and is associated with cognitive and emotional regulation [19]. Higher HRV is associated with enhanced prefrontal cortex function, which enhances EF [20, 21]. For example, studies in typically developing children have demonstrated direct correlations between higher RMSSD and improved cognitive flexibility (e.g., task-switching performance) and inhibitory control [22, 23]. Accumulating evidence has shown that physical exercise may increase HRV by enhancing parasympathetic tone, which in turn facilitates cognitive improvements in children with typical development [22–25]. Given the lower baseline HRV observed in children with ASD [26, 27], it is hoped that physical exercise could also improve EF in children with ASD by increasing their HRV. However, no previous studies have examined this aspect. Therefore, the secondary objective of the present study was to fill this research gap. In this study, we investigated this potential mediating role of HRV in the relationship between cycling interventions and EF improvements. All EF components (planning, working memory, inhibition, flexibility) and HRV were assessed at baseline (T1), mid-intervention (T2), and post-intervention (T3) over a 2-week period. We hypothesized the LTB group would show the greatest EF and HRV improvements, followed by BT and TW, with SC showing minimal changes. We expected HRV increases to mediate EF improvements, particularly in the LTB group that engages both spatial updating and dynamic balancing.

## Methods

### Study Design

**Fig. 1:**
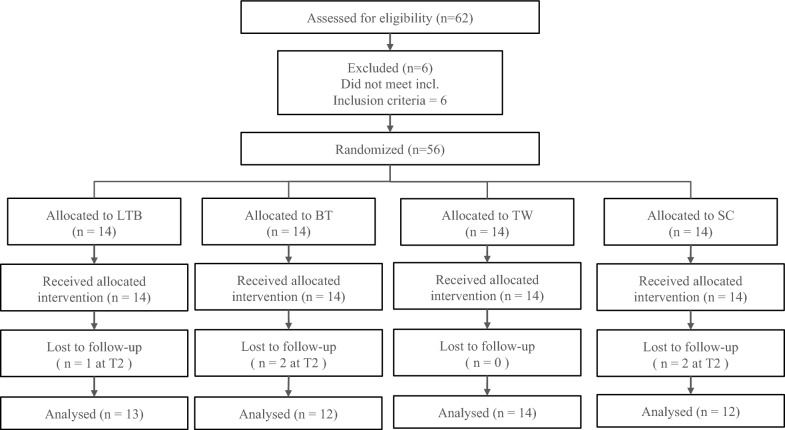
CONSORT Flow diagram of the study

This study was a four-arm randomized controlled trial (RCT) with equal allocation to three intervention groups—learning to bicycle (LTB), bicycle treadmill (BT), cycling with training wheels (TW)—and one active control group (stationary cycling, SC). Assessments were conducted at baseline (T1), mid-intervention (T2, one week), and post-intervention (T3, 2 weeks) to evaluate executive function (EF) and HRV. The study followed CONSORT guidelines (Fig. 1).

Sample Size Calculation Our previous study examining the impact of cycling training on EF in children with ASD [11] showed that the learning to ride a bicycle group had a large pre-post difference (corresponding to a Cohen’s d of 0.74) on the EF associated with planning, while the stationary cycling group had no effect (i.e., d = 0). As such, a sample of 30 participants per group is required to detect a significant difference between the learning to ride a bicycle group and the stationary cycling group with a power of 80% and an alpha of 5%. This sample size could achieve a power of 80% to detect a HRV mediation effect with paths of moderate effect size [28].

### Data Collection

Each participant was asked to perform a set of neuropsychological assessments, where different EF components were recorded. Each assessment lasted for 15 min followed by a 5-min break to prevent cognitive fatigue in the participants. The neuropsychological assessments were conducted one hour before the commencement of the first exercise intervention (T1: baseline), the 4th intervention (T2: mid-intervention) and the last exercise intervention (T3: post-intervention). The mid-assessment provides valuable information about how much variance in EF is accounted for by variance in HRV records. The HRV assessment was conducted 10 min after the end of the first (T1), 4th (T2), and last (T3) interventions using Garmin Venu 2S watches, a wrist-based device validated for short-term HRV recordings [29]. Participants sat quietly for a standardized 5-min seated rest period in a controlled, quiet environment. Within this 5-min window, the on-demand Garmin Health Snapshot feature automatically recorded a 2-min segment of inter-beat intervals to derive RMSSD. The remaining time served as a transition and stabilization period prior to and following the automated recording. Post-exercise HRV measurement was chosen to capture parasympathetic recovery, which has been shown in pediatric exercise literature to correlate more strongly with hsubsequent cognitive gains than pre-exercise resting values alone [24, 25]. During a 5 min seated rest period in a quiet environment, participants were asked to perform an on-demand Health Snapshot test (2 min) to capture parasympathetic recovery post-exercise. To ensure comfort and data quality, children were instructed to wear the Garmin Venu 2 watch only during the rest period and were asked to remain still and breathe naturally. The Garmin Venu 2’s Elevate V4 optical heart rate sensor recorded inter-beat interval data, which were processed by the Garmin Connect app to compute RMSSD [30]. Validation studies, including peer-reviewed work on similar Garmin devices (e.g., Vivoactive 4), have shown moderate-to-high agreement for RMSSD during resting conditions (e.g., concordance correlation coefficient ρc = 0.66 for RMSSD in 30-min recordings) [31]. However, the Health Snapshot feature provides a 2 min snapshot, which may limit precision compared to longer ECG recordings. Post-exercise measurement was chosen to capture parasympathetic recovery, as exercise-induced HRV changes are linked to cognitive benefits [24, 25]. These procedures were standardized across groups to minimize variability due to environmental or procedural factors.

### Participants

Sixty-two participants with mild to moderate ASD, aged 8–12 years, were recruited from a special school for intellectual disabilities, Chinese YMCA of Hong Kong and a cycling company in Hong Kong with existing research links to the study team. Inclusion criteria were: (1) ASD diagnosis per DSM-5 [32] and ADOS-2 [4]; (2) non-verbal IQ > 40 (Wechsler Intelligence Scale for Children revised in China [33]); (3) ability to follow instructions and perform interventions/assessments with assistance; (4) no additional regular physical activity (beyond school physical education) for 2 months prior; and (5) novice at two-wheel bicycle riding (< 10 s independent riding). Exclusion criteria included: (1) color blindness; (2) medical conditions limiting exercise (e.g., asthma, seizures); (3) history of brain injury or complex neurologic disorders and (4) obesity [> 95th percentile BMI [34]]. Screening was conducted by a psychologist and a psychiatric physician, with parent-reported autistic traits assessed via the Social Responsiveness Scale, Second Edition (SRS-2) [35]. In addition to this formal screening, information such as records in after-school group therapy (e.g., occupational therapy, speech therapy), medication usage and sedentary time per day were also collected from the children’s parents. After screening, fifty-six eligible participants were randomized to one of four groups using block randomization (block size = 4) to ensure equal allocation. Upon the completion of the study, participant numbers per group were: LTB (n = 13, 1 dropout at T2), BT (n = 12, 2 dropouts at T2), TW (n = 14), and SC (n = 12, 2 dropouts at T2). The study was approved by the university ethics committee. Demographic data of the participants is shown in Table 1.

**Table 1:** Demographics of participants (n = 51)

	Learning to Bicycle (LTB) (n = 13)	Bicycle Treadmill (BT) (n = 12)	Cycling with Training Wheel (TW) (n = 14)	Stationary Cycling (SC) (n = 12)	*p*
Gender	11 boys and 2 girls	10 boys and 2 girls	12 boys and 2 girls	10 boys and 2 girls	
Age (years)	8.65 ± 0.75	8.46 ± 0.84	8.96 ± 0.75	9.04 ± 0.81	.23
Weight (kg)	38.37 ± 7.72	35.52 ± 7.79	35.76 ± 7.52	37.95 ± 8.77	.73
Height (m)	1.39 ± 0.09	1.37 ± 0.12	1.41 ± 0.15	1.40 ± 0.16	.70
BMI (kg/m^2^)	19.63 ± 2.03	18.78 ± 2.24	18.15 ± 2.71	19.15 ± 3.12	.50
Non-verbal IQ	56.69 ± 7.36	56.00 ± 4.97	58.29 ± 9.27	57.08 ± 6.72	.88
SRS-2 Raw-scores	68.77 ± 18.35	69.00 ± 8.84	71.79 ± 6.07	73.33 ± 9.80	.72
Sedentary Time (min/day)	498 ± 58	502 ± 62	494 ± 65	505 ± 59	.89
Medication (n)					
Yes	2	2	2	2	
No	11	9	12	10	

### Blinding

Randomization was performed by a trained research staff in blocks of 4. Meanwhile, research staff who conducted cognitive assessments, self-regulation measurements, and data analyses were blinded to group assignments.

### Interventions

All interventions lasted for 2 weeks, comprising 8 sessions (4 sessions per week, 45 min each) in school halls/gymnasiums, led by a qualified cycling coach (Hong Kong Cycling Association) assisted by student helpers (1:1 staff-to-participant ratio). Each intervention session consisted of a 10-min preparation and warm-up, 30-min cycling training, and 5-min cool-down. Exercise intensity was monitored by asking participants every 10 min during exercise to indicate their ratings of perceived exertion (target range: 3–5) [36]. Cycling distance was measured by speedometers for non-stationary groups (LTB, TW) to ensure equivalent spatial exposure. Participants were positively reinforced verbally with compliments for their efforts during the interventions and their improvements were provided visually through graphs kept in the child’s bedroom at home (e.g., list of achievements: distance; able to use another set of wheels and able to self-launch) [37]. For the stationary cycling group, participants were provided a graph of the cumulative total time spent cycling or the equivalent distance. Apart from the LTB group, all participants in the other groups were taught how to ride a bicycle after T3 to recognize their contributions in the study.

Learning to Bicycle (LTB): Participants were asked to ride on a normal bicycle with training wheels initially. Participants then progressed to a two-wheel bicycle [see [37] for more detail]. To keep participants on the learning curve, in case the participants had successfully mastered the cycling skill before the end of the intervention period, they were asked to ride through an obstacle course that was progressively more difficult to navigate. Six of the participants in LTB group progressed to the obstacle course by session 5. The obstacle course was designed by a focus group, which consisted of four physical education teachers from participating schools and one experienced cycling coach with more than 5 years of coaching experience.

Bicycle Treadmill (BT): Participants in this group first learned how to ride a normal bicycle with its back wheel and training wheels on a roller and the front wheel on the holder stand. Then they progressed to ride the two-wheel bicycle with its back wheel on a roller (no training wheels) and the front wheel on a support stand, and finally to the conventional two-wheel bicycle with front and back wheels on the rollers.

Cycling with Training Wheels (TW): Participants in this group were asked to ride a bicycle with training wheels throughout the whole study. They also rode through an obstacle course as they became more proficient at riding.

Stationary Cycling (SC): Participants in this group were asked to cycle on a stationary bicycle, with progress tracked via cumulative time/distance graphs.

### Outcome Measures

Before the initial assessment, parents of the participants were asked to provide demographic data and a brief developmental history. Participants were then tested at T1, T2 and T3, where the measurements of EF and HRV were carried out. Each participant was asked to complete each task administered by a trained research assistant and all the tests were administered by the same research staff for consistency. Assessments were conducted at T1, T2, and T3 in participants’ schools.

Planning: Participant’s ability to plan was assessed using computerized PEBL Tower of London (PEBL TOL) task [38]. Participants were presented a sequence of three different colored balls on three pegs of different lengths on a computer screen. During each of the 12 trials, participants were instructed to move the balls using a computer mouse to match the target peg arrangement in a minimum amount of moves according to the pre-specified rules. The level of the task difficulty and the restriction on number of moves were progressively higher over time. If there were three consecutive failed trial attempts, the task was halted. Scores for children’s general planning ability were calculated by summing the correct tasks performed out of the 12 trials given, and the total score ranges from 0 to 36 [39]. Higher scores indicated better planning.

Working memory: Participants’ visual-spatial working memory was evaluated using the Corsi block tapping task (CBTT) [40]. In the CBTT, participants observed and replicated a sequence of tapped blocks, starting with three blocks. Sequence length increased by one after every two trials. The task ended after two incorrect sequences of the same length, with the longest correctly repeated sequence recorded as the score. Higher scores indicated better working memory.

Inhibition: The participants’ ability to inhibit unwanted responses to changing stimuli was measured by a computerized Go/No-go (GNG) task and was identical to the one previously [11, 41]. In the task, participants pressed a left or right key for corresponding arrows (Go response) but withheld responses for up arrows (No-go response). After 20 practice trials, they completed 300 trials: 220 Go trials (110 left, 110 right) and 80 No-go trials (26.7%), which were presented randomly via E-Prime 3.0 software [42]. Each stimulus appeared for 500 ms, followed by a 1000 ms blank interval. Breaks of 2 min were offered after every 60 trials. No feedback was provided, and response times were recorded but not analyzed due to unreliable recording [11]. False alarms (FA), defined as Go responses on No-go trials, measured inhibition, with lower FA rates indicating better inhibitory control [43].

Flexibility: Children’s Color Trail Making Test (CCTT) [44] was employed to assess cognitive flexibility. Participants completed two parts of the CCTT as quickly and accurately as possible. In Part 1 (CCTT-1), they connected numbered circles in ascending order using a single color (e.g., black 1–2-3). In Part 2 (CCTT-2), they alternated between connecting numbered circles of two colors (e.g., black 1, yellow 2, pink 3), requiring task-switching between color sets [45]. Cognitive flexibility was quantified by the interference score, which was calculated by the difference of time between Part 2 and Part 1 (i.e. CCTT-2 minus CCTT-1), where lower scores indicate better cognitive flexibility, reflecting enhanced ability to shift between mental sets.

HRV: HRV was recorded using Garmin Venu 2S wrist-based wearable watches and was expressed in terms of RMSSD.

### Statistical Analysis

All statistical analyses were conducted using SPSS (version 29.0) for Windows (SPSS Inc, Chicago, IL, USA). All the data were entered into SPSS by a research assistant. Mixed analyses of variance (ANOVA) with repeated measures were used to assess group (LTB, BT, TW, SC), time (T1, T2, T3), and group × time interactions for each of the EF outcomes (TOL, CBTT, GNG, CCTT) and HRV (RMSSD). Bonferroni correction was used to adjust the alpha levels. Generalized estimating equations (GEE) were used to examine the effects of the physical exercise interventions, the time effect, and their interaction with EF outcomes and HRV outcomes, controlling for baseline sedentary time and demographic covariates (age, sex, BMI, sedentary time). An exchangeable correlation structure was used and effect sizes were evaluated with absolute correlation or Cohen’s d > 0.2 [46]. Mediation analysis was conducted to examine the mediating effect of HRV at T2 on the relation between physical exercise and EF at T3. Preacher and Hayes’ bootstrapping [47] and conditional process modeling [48] were used to determine the significance of the mediating effect. A *p* < 0.05 was considered statistically significant. Both proposal analysis and intention-to-treat analysis (ITT) were conducted. Statistical significance was set at *p* < 0.05.

## Results

All groups successfully completed the intervention comprising 8 sessions with a total cycling time of 360 min. Following the intervention, 10 of the 13 participants in the LTB group achieved independent cycling for more than 30 s, whereas the TW and BT groups emphasized proficiency under assisted conditions. Changes in all dependent variables across the assessment time points are presented in Table 2. At baseline (T1), no significant differences were observed between groups for any outcome measures (all *p* > 0.05).

**Table 2:** Comparisons of all outcome measures between groups and within groups at different timeslots

Neuropsychological assessment	Learning to Bicycle (SD)	Bicycle Treadmill (SD)	Cycling with Training Wheel (SD)	Stationary Cycling (SD)	*p* (group effect)	*p* (interaction effect)
Planning (TOL Score)						< 0.001*
T1	4.15 (2.30)	3.00 (1.81)	4.50 (4.03)	3.75 (1.86)	0.55	
T2	6.69 (2.50)	2.75 (1.54)	6.00 (3.11)	3.25 (1.54)	< 0.001*	
T3	8.08 (2.56)	3.05 (1.79)	8.36 (3.15)	3.75 (2.60)	< 0.001*	
*p* (time effect)	< 0.001*	0.86	< 0.001*	0.66		
Cohen’s *d* effect size (95% CI)	1.74 (0.88, 2.60)	0.00 (− 0.57, 0.57)	1.41 (0.67, 2.15)	0.01 (− 0.58, 0.58)		
Working Memory (CBTT Sequence Score)						< 0.001*
T1	3.23 (0.73)	3.25 (0.45)	3.50 (0.94)	3.08 (0.67)	0.53	
T2	4.62 (0.51)	3.42 (0.67)	4.79 (0.89)	3.33 (0.89)	< 0.001*	
T3	4.92 (0.49)	3.00 (0.74)	4.93 (0.73)	3.42 (0.67)	< 0.001*	
*p* (time effect)	< 0.001*	0.06	< 0.001*	0.20		
Cohen’s *d* effect size (95% CI)	2.25 (1.23, 3.28)	0.40 (− 0.19, 0.99)	1.68 (0.86, 2.49)	0.51 (− 0.09, 1.11)		
Inhibition (GNG FA Error)						0.036*
T1	20.31 (2.29)	21.33 (1.37)	20.14 (4.22)	21.00 (2.86)	0.70	
T2	17.00 (3.08)	20.75 (2.14)	19.43 (3.39)	20.25 (2.42)	0.001*	
T3	16.23 (2.86)	18.08 (3.78)	18.79 (2.58)	19.33 (2.50)	0.06*	
*p* (time effect)	< 0.001*	< 0.001*	0.03*	0.006*		
Cohen’s *d* effect size (95% CI)	1.55 (0.74, 2.36)	0.97 (0.29, 1.66)	0.56 (− 0.01, 1.12)	0.59 (0.01, 1.17)		
Flexibility (CCTT Interference Score)						< 0.001*
T1	41.85 (6.59)	44.92 (6.58)	44.93 (9.58)	44.75 (8.34)	0.70	
T2	38.15 (5.08)	42.91 (7.03)	40.79 (7.89)	43.58 (7.51)	0.21	
T3	34.00 (5.74)	44.75 (6.74)	37.07 (7.89)	42.67 (7.11)	0.001*	
*p* (time effect)	< 0.001*	0.06	< 0.001*	0.16		
Cohen’s d effect size (95% CI)	1.17 (0.58, 1.76)	0.07(− 0.50, 0.63)	0.79 (0.24, 1.34)	0.10 (− 0.47, 0.67)		
HRV (RMSSD, ms)						0.05
T1	36.95 (3.58)	39.52 (5.20)	41.03 (5.23)	37.82 (5.05)	0.15	
T2	41.87 (3.27)	40.44 (5.47)	42.77 (6.97)	41.59 (3.97)	0.61	
T3	43.42 (5.67)	39.62 (4.78)	45.63 (7.52)	39.70 (4.25)	0.29	
*p* (time effect)	< 0.001*	0.51	0.69	0.06		
Cohen’s d effect size (95% CI)	1.24 (0.52, 1.96)	0.03 (− 0.54, 0.60)	0.06 (− 0.46, 0.59)	0.30 (− 0.28, 0.87)		

TOL = Tower of London; CBTT = Corsi Block Tapping Task; GNG FA error = Go/No-go False Alarm error; CCTT = Children’s Color Trails Test interference score (CCTT-2 minus CCTT-1, in seconds). Lower CCTT and GNG scores indicate better performance; higher TOL and CBTT scores indicate better performance. HRV = Heart Rate Variability (RMSSD, root mean square of successive differences, in milliseconds). Higher HRV values indicate greater autonomic flexibility. *p* < .05 indicates statistical significance

### Planning (TOL Score)

A significant group × time interaction was observed [F (6,94) = 7.28, *p* < 0.001]. Significant group effects were observed at mid-intervention (T2) and post-intervention (T3) (ps < 0.001). LTB and TW outperformed BT and SC (ps < 0.05) at both timeslots. Within-group analyses showed significant time effects for LTB [*p* < 0.001, d = 1.74, 95% CI (0.88, 2.60)] and TW [*p* < 0.001, d = 1.41, 95% CI (0.67, 2.15)], but not for BT and SC (ps > 0.05). GEE analyses revealed significant intervention effects for LTB (β = 3.93, *p* < 0.001) and TW (β = 3.86, *p* < 0.001) with moderate-to-large effect sizes (d’s > 0.2). Mediation analysis showed that HRV partially mediated the intervention effect on TOL scores in the LTB group [indirect effect: β = 0.42, *p* = 0.04, 95% CI (0.02, 0.82)], but not in TW, BT and SC (ps > 0.05).

### Working Memory (CBTT Sequence Score)

A significant group × time interaction was observed [F (6,94) = 6.91, *p* < 0.001]. Significant group effects were shown at T2 and T3 (ps < 0.001). LTB and TW outperformed BT and SC (ps < 0.05) at both timeslots. Within-group comparisons showed significant time effects in LTB [*p* < 0.001, d = 2.25, 95% CI (1.23, 3.28)] and TW [*p* < 0.001, d = 1.68, 95% CI (0.86, 2.49)], but not in BT and SC (ps > 0.05). GEE analyses showed significant intervention effects for LTB (β = 1.69, *p* < 0.001) and TW (β = 1.43, *p* < 0.001) with large effect sizes (d’s > 1.4). Mediation analysis showed that HRV partially mediated the intervention effect on CBTT scores in the LTB group [indirect effect: β = 0.35, *p* = 0.03, 95% CI (0.03, 0.70)] and TW group [indirect effect: β = 0.31, *p* = 0.04, 95% CI (0.02, 0.65)], but not in BT and SC (ps > 0.05).

### Inhibition (GNG FA Error)

A significant group × time interaction was observed [F (6,94) = 3.45, *p* = 0.004]. Significant group effects were found at T2 and T3 (ps < 0.05). LTB, BT and TW showed fewer errors than SC (ps < 0.05) at both timeslots. Within-group time effects were significant for all groups: LTB [*p* < 0.001, d = 1.55, 95% CI (0.74, 2.36)], BT [*p* <  = 0.001, d = 0.97, 95% CI (0.29, 1.66)], TW [*p* = 0.03, d = 0.56, 95% CI (− 0.01, 1.12)], and SC [p = 0.006, d = 0.59, 95% CI (0.01, 1.17)]. GEE analyses revealed significant intervention effects for LTB (β = − 3.76, *p* = 0.003) and BT (β = − 2.17, *p* = 0.01) with moderate to large effect sizes (d’s > 0.56). Mediation analysis showed that HRV partially mediated the intervention effect on GNG FA errors in the LTB [indirect effect: β = − 0.48, *p* = 0.04, 95% CI (− 0.92, − 0.04)] and BT group [indirect effect: β = − 0.35, *p* = 0.045, 95% CI (− 0.70, − 0.01)], but not in the other two groups (ps > 0.42).

### Flexibility (CCTT Interference Score)

A significant group × time interaction was observed [F (6,94) = 4.56, *p* < 0.001]. Significant group effects were shown at T3 (*p* < 0.001). LTB and TW had lower scores than BT and SC at T3 (ps < 0.05). Within-group time effects were significant for LTB [*p* < 0.001, d = 1.17, 95% CI (0.58, 1.76)] and TW [*p* < 0.001, d = 0.79, 95% CI (0.24, 1.34)], but not in BT and SC (ps > 0.05). GEE analyses indicated significant intervention effects for LTB (β = − 7.85, *p* < 0.001) and TW (β = − 7.86, *p* < 0.001) with moderate to large effect sizes (d’s > 0.07). Mediation analysis showed that HRV partially mediated the intervention effect on CCTT scores in LTB [indirect effect: β = − 0.48, *p* = 0.02, 95% CI (− 0.89, − 0.07)] and TW [indirect effect: β = − 0.39, *p* = 0.03, 95% CI (− 0.75, − 0.03)], but not in the other two groups (ps > 0.05).

### Heart Rate Variability (RMSSD)

A marginal significant group × time interaction was observed [F (6,94) = 1.53, *p* = 0.05], and no significant group effects were shown at any timeslots (ps > 0.05). Within-group analyses showed a significant time effect for LTB [*p* < 0.001, d = 1.24, 95% CI (0.52, 1.96)] but not in the other groups (ps > 0.05). GEE analyses indicated significant intervention effects for LTB (β = 6.22, *p* < 0.001) with a large effect size (d > 1.2).

### Mediation Analyses

As shown in Table 3, results revealed that HRV at mid-intervention (T2) partially mediated the intervention effects on EF outcomes at post-intervention (T3) in both LTB and TW groups, but not in BT or SC groups. Specifically, HRV at T2 significantly mediated working memory (LTB: β = 0.35, TW: β = 0.31) and flexibility (LTB: β = − 0.48, TW: β = − 0.39). Moreover, HRV significantly mediated planning (β = 0.42) and inhibition (β = − 0.48) in the LTB group.

**Table 3:** Summary of mediation analyses (HRV at T2 mediating intervention effects on EF at T3)

Intervention Group	Executive Function Domain	Total Effect (c)	Direct Effect (c')	Indirect Effect (a × b) [95% CI]	Path a (Intervention → HRV at T2)	Path b (HRV at T2 → EF at T3)
LTB	Planning (TOL score)	3.93***	3.51***	0.42* [0.02, 0.82]	4.92	0.085
LTB	Working memory (CBTT)	1.69***	1.34***	0.35* [0.03, 0.70]	4.92	0.071
LTB	Inhibition (GNG false alarms)	− 3.76***	− 3.28***	− 0.48* [− 0.92, − 0.04]	4.92	− 0.098
LTB	Flexibility (CCTT interference)	− 7.85***	− 7.37***	− 0.48* [− 0.89, − 0.07]	4.92	− 0.098
TW	Planning (TOL score)	3.86***	3.86***	ns	1.74	–
TW	Working memory (CBTT)	1.43***	1.12***	0.31* [0.02, 0.65]	1.74	0.178
TW	Inhibition (GNG false alarms)	ns	ns	ns	1.74	–
TW	Flexibility (CCTT interference)	− 7.86***	− 7.47***	− 0.39* [− 0.75, − 0.03]	1.74	− 0.224
BT	Planning (TOL score)	ns	ns	ns	0.92	–
BT	Working memory (CBTT)	ns	ns	ns	0.92	–
BT	Inhibition (GNG false alarms)	–2.17**	–1.82**	–0.35* [–0.70, –0.01]	0.92	− 0.380
BT	Flexibility (CCTT interference)	ns	ns	ns	0.92	–
SC	Planning (TOL score)	ns	ns	ns	3.77	–
SC	Working memory (CBTT)	ns	ns	ns	3.77	–
SC	Inhibition (GNG false alarms)	ns	ns	ns	3.77	–
SC	Flexibility (CCTT interference)	ns	ns	ns	3.77	–

*p* < .05, ** *p* < .01, *** *p* < .001 (based on bootstrapped confidence intervals or original GEE p-values reported in text). ns = not statistically significant (*p* ≥ .05). – = not applicable (mediation path not tested due to non-significant indirect effect). Path a represents the estimated change in RMSSD (ms) from T1 to T2 attributable to the intervention (derived from within-group mean differences in Table 2). Path b represents the estimated change in the executive function outcome per 1 ms increase in RMSSD at T2. Lower scores on GNG false alarms and CCTT interference indicate better performance; therefore, negative beta coefficients for these domains reflect improvement. All mediation analyses were conducted using bootstrapped estimates (Preacher & Hayes, 2004; Hayes, 2017). Abbreviations: LTB = Learning to Bicycle; TW = Training Wheels; BT = Bicycle Treadmill; SC = Stationary Cycling; TOL = Tower of London; CBTT = Corsi Block-Tapping Task; GNG = Go/No-Go; CCTT = Children’s Color Trails Test; HRV = heart rate variability; RMSSD = root mean square of successive differences

## Discussion

The present study examined the effects of four cycling interventions on executive function (EF) in children with ASD through heart rate variability (HRV, RMSSD) mediation. The primary findings showed that all EF components improved significantly in both the learning-to-bicycle (LTB) and training-wheels (TW) groups but not in the bicycle-treadmill (BT) or stationary-cycling (SC) groups. These findings are consistent with previous research showing that cognitively-engaging exercise interventions enhanced EF in children with ASD [10, 11, 49].

Although all EF improvements were evident in both LTB and TW groups, it is worthwhile to explore the differences between the two groups. Results showed that the LTB group exhibited larger effect sizes than the TW group. This may be due to greater cognitive load from simultaneous navigation and unsupported balance. TW group may have reduced balance demands but still required some postural control, which therefore made it a primarily spatial-updating intervention and not purely isolated. Meanwhile, one of the objectives in the present study was to investigate the role of two distinct components in cycling, namely spatial updating and dynamic balance, in driving the positive impacts of bicycling on EF. Comparing the neuropsychological performance between the LTB and TW group, the LTB group exhibited larger effect sizes for all the EF improvements than the TW group. This may be due to the differences in cognitive loading between the groups. The LTB intervention required participants to navigate and maintain balance simultaneously (i.e., engaging both spatial updating and dynamic balance components) during the performance. In contrast, the TW group focused primarily on navigation (i.e., spatial updating) with a reduced load for maintaining balance due to the training wheels (i.e., requiring less dynamic balance). As a result, the LTB group demanded greater cognitive resources than the TW group and yielded more substantial EF improvements.

Given the primary objective of the present study to investigate the roles of spatial updating and dynamic balance components in enhancing EF, it is also important to examine the neuropsychological differences between the TW and BT groups. These groups were specifically designed to isolate one component each: dynamic balance for BT and spatial updating for TW. Interestingly, the BT group only showed a significant improvement in inhibition but no significant changes in other EF domains. This selective enhancement may be due to the nature of the task, during which participants were required to intensively focus on maintaining balance while suppressing extraneous thoughts or distractions—potentially compounded by the short intervention duration, which may not have allowed sufficient time for the balance demands to fully recruit processes driving changes in the other EF domains. In this regard, the TW group started navigating almost immediately, whereas the BT group would only have really started to balance fully toward the end of the intervention, making the BT experience similar to the SC group for much of the period; we might have seen broader differences in BT with a longer intervention. Consequently, inhibitory control was enhanced in BT, aligning with previous studies showing that balance-oriented exercises, which require sustained attention and motor precision, can selectively augment inhibitory processes in children with ASD [50]. In contrast, the TW intervention yielded broader gains in all EF domains (e.g., planning, working memory, flexibility and inhibition). One possible explanation may lie on the cognitive demands of spatial updating, which required participants to anticipate paths, memorize obstacle position and cycling route, adapt to environmental changes and override impulsive responses. All these processes mirrored real-world demands on all EF domains. The comparisons between the BT and TW groups have provided intriguing evidence for a potential matching relationship between exercise components and specific EF domains, which suggested that tailored motor-cognitive integrations (e.g., combining balance for inhibition or navigation for multi-domain gains) may optimize interventions for children with ASD.

One of the particular strengths of the present study was the investigation of the potentially mediating role of HRV between interventions and EF domains. As shown in Table 3, mediation analyses revealed that HRV at mid-intervention (T2) partially mediated the intervention effects on EF outcomes at post-intervention (T3) in both LTB and TW groups and the HRV significantly mediated planning and inhibition in the LTB group. These findings have two major implications. First, they have extended the established link between HRV and EF domains, previously demonstrated with typically developing populations (e.g., [22, 26]) to children with ASD. Second, the results suggested that early improvements in HRV may contribute to subsequent improvements in EFs, particularly in the LTB intervention that required both spatial updating and dynamic balance components. This notion is further supported by the absence of mediation in BT and SC groups, which exhibited minimal HRV and EFs improvements. Indeed, these results have underscored HRV as a potential mechanism linking physical exercise to cognitive outcomes in children with ASD.

Apart from theoretical implications, the findings of the present study also have practical value for designing exercise interventions for children with ASD. The findings from the LTB group suggest that exercise integrating balance and navigation may enhance both EF and autonomic flexibility. Moreover, results of the TW intervention have provided a viable alternative for children who may struggle with balance demands. We recommend that practitioners and parents should prioritize physical exercise with cognitive-motor components for children with ASD or for those who have cognitive deficits. Meanwhile, the present study also supports the notion of incorporating HRV monitoring (e.g., via wearable devices like Garmin Venu 2) to help track the progress of exercise programmes [51]. Cycling interventions are cost-effective and easy to implement, aligning with the need for accessible strategies to address EF deficits in children with ASD [9].

### Limitations

While the cognitive benefits of the cycling interventions are evident in the present study, there are several limitations that require attention. First, the small sample sizes (n = 12–14 per group) of the present study were inadequate to meet the required sample size (n = 30 per group), which may therefore limit the generalizability of the findings and increase variability. Thus, results should be interpreted as exploratory, and larger trials are needed to confirm findings. The reason for not recruiting more participants to replace the ineligible and drop-out participants was due to the difficulty finding more individuals who met the inclusion criteria, as well as the budgetary restrictions of the present study. Nevertheless, the effect sizes we expected to see based on our original power analysis and sample size calculation were actually much larger than anticipated in the current study. Consequently, our analyses still detected significant group and time effects as well as their interactions. Second, the short intervention duration (8 sessions) limits insights into long-term effects. It is recommended that future research should incorporate a longer period of intervention (e.g., five weeks or more). Third, the present study did not include a no-exercise control group. Including such a pure control group, as in Tse et al. [11], could clarify the specific contributions of cycling components. Fourth, HRV was measured using a 2-min post-exercise snapshot via the Garmin Venu 2S, which, while validated in conference proceedings [29] and supported by peer-reviewed studies on analogous Garmin models [31], may not capture full resting HRV dynamics due to its short duration and potential for motion artifacts. Future studies should use longer, ECG-based recordings for greater accuracy. Fifth, HRV was assessed during post-exercise recovery rather than at pre-exercise rest, while this timing was chosen to index autonomic rebound linked to cognitive adaptation, future studies should include both pre- and post-exercise measurements for direct comparison. Additionally, variability in LTB (e.g., 6/13 progressing to obstacles) may introduce heterogeneity despite this was designed to maintain challenge. Finally, it is recommended that future research should incorporate some objective measures of balance and navigation (e.g., motion capture) in order to quantify their impact on EF.

## Conclusions

The present study demonstrates that cycling interventions incorporating balance and navigation can significantly enhance EF in children with ASD, with HRV partially mediating cognitive improvements. By identifying effective intervention components, this study has shown the importance of incorporating cognitive-motor demands in exercise interventions to ameliorate EF deficits in children with ASD, with subsequent benefits for daily functioning and quality of life in the population.

## Data Availability

The data supporting the findings of this study are available from the corresponding author upon reasonable request.
